# Evolving ‘self’‐management: exploring the role of social network typologies on individual long‐term condition management

**DOI:** 10.1111/hex.12394

**Published:** 2015-08-18

**Authors:** Rebecca L. Morris, Anne Kennedy, Caroline Sanders

**Affiliations:** ^1^ Centre for Primary CareThe University of ManchesterManchesterUK; ^2^ NIHR Collaboration for Leadership in Applied Health Research (CLAHRC) WessexHealth SciencesFaculty of Health SciencesUniversity of SouthamptonSouthamptonUK

**Keywords:** long‐term conditions, network typologies, qualitative, self‐management, social networks, UK

## Abstract

**Background:**

Whilst there has been a focus on the importance of social support for managing long‐term conditions, there has been little specific focus on the characteristics of social networks that shape self‐management. Policy emphasis is placed on individual responsibility for self‐care, and this influences commissioning of health‐care services. Assumptions are often made by policymakers about accessibility and preference for support and the influence of the social context on chronic illness management.

**Objective:**

To examine the social networks of individuals with long‐term conditions and identify how the characteristics of their composition influences support needs.

**Design, setting and participants:**

Thirty participants completed initial face‐to‐face in‐depth interviews, telephone follow‐ups and final face‐to‐face interviews in the north‐west of England. A longitudinal qualitative design was used to elicit the subtle changes in relationships over a year.

**Findings:**

The findings suggest that the relationships which constitute a social network influence perceived support needs and attitudes to self‐management. The amalgamation of relationships was characterized into three network typologies (family focused, friend focused or health‐care professional focused) according to which types of relationships were dominant. In the absence of support, accounts highlighted a small number of substitutes who could provide support at times of critical need.

**Discussion:**

This study challenges the notion of ‘self’‐management as an individual construct as many of the practices of illness management involved the support and/or negotiation of roles with others. By examining the nuances of relationships, this study has highlighted the tacit boundaries of practical and emotional support provision.

## Introduction

Supporting self‐management for individuals with long‐term conditions has been a key aim of UK and global health policy. Self‐management is defined as *the actions individuals take for themselves and their families to stay healthy and to care for minor, acute and long‐term conditions*.^1^ The focus of formal self‐care support has been on developing educational materials and improving communication between patients and clinicians.^2^ Yet chronic disease self‐management programmes have been identified as not prioritizing or tailoring goals that are of importance to patients.^3^ Despite an espoused ethos of supporting a ‘social model’ of illness, in practice this is often lacking in self‐management programmes.^4^, ^5^ Examining social networks for chronic illness management is one approach that may address this shortfall. Social network research focuses on the relationships of social actors by examining systems through which social interaction occurs at the level of the individual, group or organization.^6^ This approach moves beyond the individual, examining the nuances of multiple interrelated relationships.

Conceptualizing support for self‐management as a social network moves away from the idea that an individual's set of actions and behaviours alone are responsible for sustaining health, but little is known about how changes in social networks over time influence an individual's capacity to manage their health.^7^, ^8^ Patients with chronic conditions face challenges (such as coping with symptoms) that are experienced within the contexts of formal health care, informal social network members and the physical environment.^9^ These three contexts represent distinct but overlapping spheres, influencing the timing of health‐care utilization and integration of information and support; however, these distinctions and overlaps have remained underexplored.

Within the sociology of chronic illness, families and significant others have been a reference point for day‐to‐day decision making and illness management in domestic settings.^10^ Whilst social network research has illuminated the impact of social networks on the genesis of long‐term health conditions,^11^ there has been little in‐depth research on the role of social networks in *on‐going* condition management. Reeves and colleagues^7^ found that personal networks can be a substitute for formal care. This research illuminated patterns of work within social networks, but there remains a need to understand in‐depth how this is enacted in individuals' everyday contexts. Social networks have the potential to alter the role of health‐care professionals and change patterns of health inequalities.^12^ A social network perspective of long‐term condition management is needed to enable the consideration of a wider set of relationships and a broader perspective of priorities.^8^ Despite growing evidence of the role of social networks in self‐management, there remains a gap between much of the research on self‐management support, and the everyday reality of living with a long‐term condition.^13^

Exploring the types of social networks that people with long‐term conditions have can help to situate the impact of wider contextual influences on illness management and the systems of support available.^14^ At the individual level, this has implications for the type of information and support sought. For instance, Fiori and colleagues^15^ identified how network types vary in the quality of support provided by social network members. Currently, there is a need to understand the types of networks that support, or undermine, self‐management and the properties of such networks which might be relevant in the development of new interventions.^8^, ^16^ By examining network types, emergent properties can be identified to explain management characteristics that remain intangible when focusing on their constituent parts.^17^ The aim of this study was to examine the composition of social networks for individuals with long‐term conditions and identify how the nature of networks implicated in the mundane tasks of long‐term condition management influences support needs.

## Methods

A longitudinal qualitative social network study was conducted with individuals who had a long‐term condition in the north‐west of England between 2008 and 2009. This study was embedded within a formative evaluation preceding a randomized controlled trial (RCT) aimed at implementing self‐management support for long‐term conditions.^18^, ^19^ Ethical approval for this study was granted by the Oldham Research Ethics Committee (REC reference: 07/H1011/96) (Table 1).

**Table 1:** Participant demographic information and types and amount of multiple conditions reported

Participant pseudonym	Gender	Age at start of study	Index condition	Comorbid conditions (self‐defined)	Network typology
Catherine	Female	36	IBS	Occipital neuralgia, reoccurring cystitis	Friend focused
Chris	Male	65	Diabetes	None reported	Health‐care professional focused
James	Male	59	Diabetes	High blood pressure, cholesterol	Family focused
Beatrice	Female	46	Diabetes	None reported	Family focused
Abbie	Female	53	COPD	IBS, depression	*Unknown*
Tina	Female	69	Diabetes	Stress incontinence, eating and sleeping problems, hair loss, eye infections, skin and gum infections	Family focused
Adrian	Male	82	Diabetes	Rheumatoid arthritis, high blood pressure	Family focused
Don	Male	48	Diabetes	Cataracts and eye problems, tendonitis	Family focused
Adam	Male	–	Diabetes	Knee problems, kidney problems	Family focused
Danielle	Female	66	Diabetes	MS, underactive thyroid, high cholesterol	Family focused
Natalie	Female	57	IBS	High blood pressure, cholesterol, hypertension, COPD	Family focused
Lyn	Female	57	COPD	IBS	Family focused
Leo	Male	51	IBS	None reported	Family focused
Frank	Male	65	COPD	Hypertension	Health‐care professional focused
Tom	Male	52	Diabetes	High cholesterol	Health‐care professional focused
Rachel	Female	–	COPD	None reported	Unknown
Jane	Female	55	Diabetes	Epilepsy	Family focused
Sarah	Female	31	IBS	None reported	Family focused
Debbie	Female	62	IBS	None reported	Family focused
Ron	Male	84	Diabetes	Ischaemic heart disease, arthritis	Friend focused
Ted	Male	83	IBS	Hearing problems, high cholesterol, memory problems, back pain, signs of angina (participant wording)	*Unknown*
Isabella	Female	50	Diabetes	Chronic depression	*Unknown*
Kate	Female	84	COPD	High blood pressure, blackouts	Health‐care professional focused
Nancy	Female	76	COPD	Arthritis	Family focused
Jack	Male	65	Diabetes	High blood pressure, high cholesterol	Family focused
Todd	Male	44	IBS	None reported	Family focused
Zac	Male	65	Diabetes	Heart bypass, ulcers on bottom of feet that would not heal	Health‐care professional focused
Rita	Female	25	IBS	Anxiety problems	Friend focused
Matthew	Male	69	COPD	Oesophageal problems (caused by a hiatus hernia), feet problems	Friend focused
Donna	Female	54	Diabetes	High blood pressure and high cholesterol	Family focused

COPD, chronic obstructive pulmonary disease; IBS, irritable bowel syndrome.

### Data collection

Semi‐structured interviews explored participants’ management strategies, experiences with health‐care providers and the influence of social networks on condition management. This semi‐structured approach enabled emerging areas to be discussed with participants.^20^ Participants completed a short demographic questionnaire at the beginning of the initial interview. In the final interview, we used a social network approach to gather information about the support they received and what influenced their long‐term condition management. Participants were asked to map their social network members using a diagram with three concentric circles.^21^, ^22^ Participants placed members in the central circle in response to the following question: ‘Who do you think are important to you in terms of how you manage your health and long‐term condition?’ Members placed in the middle circle were considered by participants to be less important than those in the central circle, and members in the outer circle were considered less important than those in the other circles. The centre of the social network diagram represents the participant (or ego), and the other circles represent network members (or alters). The thickness of the line between the ego and the alters represents how frequently the participant had contact with the network member (i.e. the thicker the line, the more regular the contact), and the size of the circle represents the proximity of the network member to the participant (i.e. the larger the circle, the closer the network member lives). The gender of the network member is represented inside the circle (i.e. symbols with an arrow end represent male participants, whilst symbols with a cross end represent female participants). Participants were asked about the nature of the relationships as well as changes in illness management. All interviews were conducted by RM.

### Data analysis

All initial and final interviews were audiotaped and transcribed. Field notes were made during telephone calls. The duration of initial interviews was from 34 to 92 min (average of 59 min), telephone interviews from 5 to 16 min (average of 12 min), and the final interviews from 44 to 79 min (average of 62 min). All names reported in the transcripts and network diagrams were anonymized using relationship codes and pseudonyms. Data analysis was on‐going throughout the study. Meetings with all authors were held regularly to discuss emerging themes. All authors analysed the transcripts and commented on the interpretation of the data set, key concepts and themes. In longitudinal data analysis, it is appropriate to examine both between and within cases to consider the temporal changes that reflect individual experiences.^23^ This was carried out by combining thematic and narrative analyses. Combining approaches allowed themes that emerged across the data set to be identified whilst maintaining the context of management, which was central to understanding network involvement. Atlas.ti version 5.2 (Atlas.ti, Berlin, Germany) and VennMaker version 1.03 (VennMaker, Cologne, Germany) were used to support analysis. The network diagrams were analysed in aggregate and then considered a separate unit of analysis to examine variations within an individual network.^24^ Network diagrams were analysed descriptively to identify who was in the network, and the project took an individual network approach to understand with whom the participants discussed their health and condition management and the types of support sought across the network. Network types were identified through the analysis by combining the network composition and the narrative descriptions of the meanings that participants ascribed to relationships within their network with their condition management. Each participant was considered as a case, and their narrative was used to challenge the typology characteristics to identify the boundaries of each network type.

## Results

**Figure 1:**
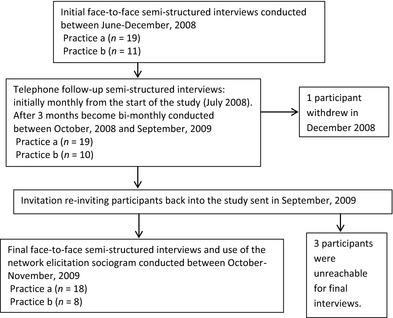
Data collection flow chart.

**Figure 2:**
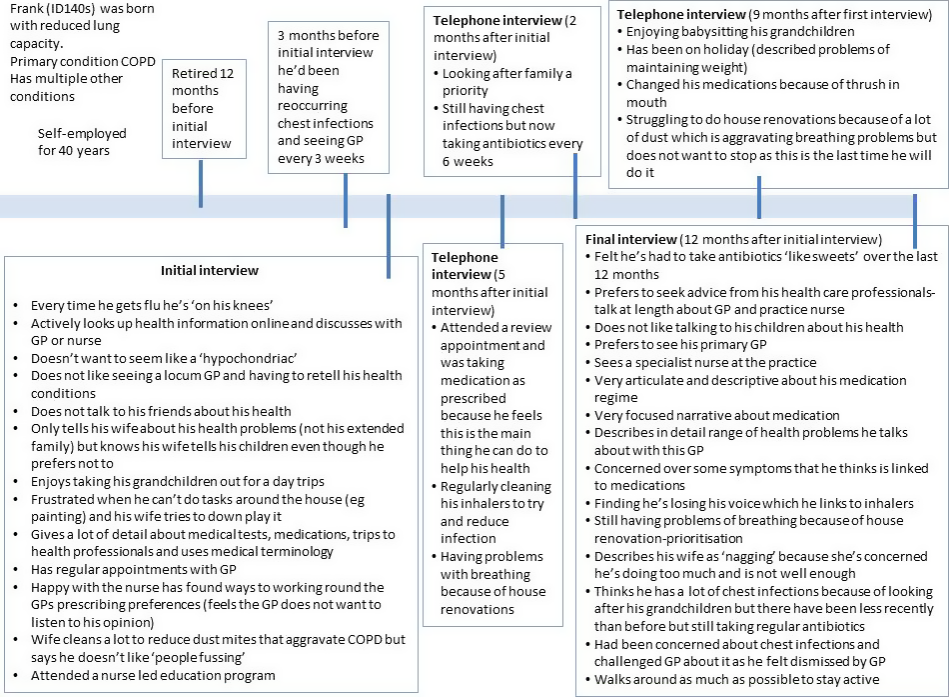
Timeline of Frank's interview data.

Thirty participants were purposefully sampled^25^ with an index condition of diabetes, chronic obstructive pulmonary disease (COPD) or irritable bowel syndrome (IBS). Nine people approached declined to participate. Participants were recruited from two general practices (Practice A *n* = 19, Practice B *n* = 11) in an economically depressed area in the north‐west of England (Fig. 1). Consent was obtained from all participants before initial interviews and reconfirmed before final interviews. One participant withdrew after 5 months because of ill health, and three were unreachable for final interviews. Initial face‐to‐face interviews (Table 2), telephone follow‐up (Table 3) and final face‐to‐face interviews a year later (Table 4) were conducted with participants (Fig. 1). Timelines were used to manage individual stories and summarize key topics and events (Fig. 2). Comorbidity also emerged as a salient issue.^26^

**Table 2:** Baseline interview guide

How would you describe your current state of health? What are the conditions you have? Which one if any has priority at the moment in terms of having to manage it?
When did you start to have contact with health services about this?
How does having your condition affect your life on a day‐to‐day basis?
How do you manage NOW on a day‐to‐day basis with your condition?
Have you had to make any changes to your lifestyle and your diet?
What do you currently do when your symptoms get worse?
Starting from when you first thought something was wrong can you tell me how you have responded to your illness and what sort of adjustments you have had to make to your life and what you do on a daily basis?
Are there things that other people tell you should be doing but you do not do? What are these things and how do you feel about other people telling you these things?
Have you used any information concerning your condition?
Do you speak to the pharmacist at all about medication for long‐term conditions?
Contact with voluntary organizations concerned with your condition?
Who in your family or circle of friends locally do you talk to about your illness? When and what do you talk about?
Are there occasions when you prefer not to talk to people and keep things to yourself and if so why?
What things in your neighbourhood make it easier to manage having a long‐term condition and what things make it difficult?
What sorts of contact with people (your family and friends, neighbours, local people) make things easier in managing a long‐term condition and what makes things more difficult for you?
How long have you lived in the area?
Could you describe your neighbourhood to someone who was not from the area?
Do you feel part of a community?
Do you get on with/look out for your neighbours and vice versa?
Would you say you know most of the people in your neighbourhood?
Does anyone help you? If so in what ways?
How many times in the past 2 weeks have friends or family visited you or you visited them?
Who have you talked to about your health?

**Table 3:** Monthly interview guide

How would you describe you current state of health?
Have there been any changes in your condition in the last month? Have you had any time off work?
Has your condition changed your life on a day‐to‐day basis in the past month? What has having your condition changed in your life?
Have you made any special changes to your diet or lifestyle in the past month?
What prompted changes? Have you read anything/seen on TV/Internet?
Are you able to exercise regularly? What helps/hinders?
What health services have you used in the last few weeks?
Who have you spoken to? E.g. pharmacist, NHS direct, smoking cessation programme, condition specific clinic?
Have you made any appointments?
Who have you asked for help from in your family or friends? If so, who, how regularly and what have they been doing? Have you visited any friend or family in the past month or had them visit you? If so, who, how often and where did you meet?
Who you spoken to any friends or family about your condition?
Have you been in contact with any voluntary organizations concerned with your conditions? Have you received information or support from any other sources for your condition?
Is there anything important, that we have not discussed, that has happened in the last month that has affected your health which you would like to mention?

**Table 4:** Final interview guide

In the inner circle place those who are most important to you in terms of your health, in the next circle place those who are important but not quite as important as those in the inner circle, in the outer circle places those who are important but not as important as those in the other circles
Highlight in the same colour the network members who know each other
How far away do they live/work (write next to name):
1a: co‐habiting
1b: short walk/drive away
1c: lives up to 1 h away
1d: over 1 h away
How much contact do you have with them (write next to name):
2a: daily
2b: at least once a week
2c: at least once a month
2d: every couple of months
2e: less often than every couple of months
Who is most important in the network for you? Why?
Compare the different network members and what they mean/their role in management
Who among the people in your diagram do you help? Why?
Was there anyone who was more important but is less so now?
Is there anyone who was less important but is more important now? Why?
Are there people who are making it difficult in some ways? some people have said that there are certain people that make it harder for them to manage their condition, is there anyone like that in your diagram?
Who do you socialize with? Are their people who are not on here who you see regularly? Would you talk to them about your health?
Out of these who would you talk to about health issues/ask for help? Is this different to the people you spend time with or talk to generally?
Health‐care professionals: Did they mention or not? How do you feel about your relationship? How do you prioritize their role in your health care?
Who do you talk to about your condition? How often? Has this changed over time? How is this different to the people that you are close to or spend a lot of time with generally do you talk to them about your health? If no, why?
What is your main condition priority at the moment? How has your condition(s) changed over the last year?
What things have stayed the same, got better or changed for you over the last X months
Have you *done* anything differently about your condition over the last x months?
Have you changed the way *you think* about your condition and in what way?
New things you have done: Have you talked to anyone or made contact with any service or local activity over the last year?
Have you stopped seeing anyone or doing things you previously did at work home or locally? If so, what are the reasons?
More broadly how has the area changed? What is the most important thing in the area that you use and things that you do daily activities? How have these changed? Has this been effected by your health?
Tell me about your contact with primary care over the last few months. For your chronic condition, for other things? Has it changed over the last year? If so, how is it different from before? Has this impacted on how you view your condition and support from the service and elsewhere?
How relevant or important has this change been compared to other changes for you in living with your condition over the last year?
Have you noticed any differences in the priorities of the GP or nurse during the consultations?
How often do you see the GP or nurse in the last 12 months?
In general has anything changed for you in the way in which you use or talk to people in primary care about your chronic condition?
Have you noticed any differences in the way that primary care responds to you or your chronic condition and generally?
If have more than one condition – Did you focus on one condition over the other during your contact with services? Thinking about who you have spoken to about your health, would you talk to them about both of your conditions? Is there any difference in which/what you would talk about?
Are you involved with any voluntary organizations? Have you joined anything locally over the last year? Do you do anything differently in the way in which you talk to people about your illness or what you do on a daily basis including in the work place?
What has been the most significant change for you over the last year?

The social networks of participants comprised a wide variety of members, including partners, family members, pets and health‐care professionals (Table 5). The three types of networks identified are characterized by differing combinations of features which influenced management priorities, and the degree to which they facilitated normalization (i.e. incorporation into everyday routines) of illness management (Table 6):

**Table 5:** The total number of each network member placed in the network elicitation diagram per category of importance

Most important category (inner circle)		Important category (middle circle)		Less important category (outer circle)	
Children	18	Friend	20	Friend	20
Partner	15	Work colleague	13	Niece/nephew	6
Friend	13	Neighbour	12	Niece/nephew‐in‐law	4
Sibling	12	Children	9	Nurse	3
Grandchildren	10	GP/doctor	8	Work colleague	3
Pets	8	Specialist/surgeon	5	Sibling	3
GP/Doctor	7	Grandchildren	4	Cousin	3
Parent (mother *n* = 6, father *n* = 1)	7	Mother	3	Grand‐nephew	3
Children‐in‐law	5	Sibling	3	Pharmacist	2
Nurse	4	Partner	3	Neighbour	2
Hospital	2	Nurse	3	Podiatrist	2
Step‐children	1	Children‐in‐law	2	Sibling‐in‐law	2
Ex‐wife	1	Cousin	2	Children	2
Parent‐in‐law	2	Aunt	1	Herbalist	1
Podiatrist	1	Cousin‐in‐law	1	Dog	1
Alternative therapist	1	Organizer	1	Staff at GP surgery	1
Friend (deceased)	1	Specialist clinic	1	Church group	1
Counsellor	1			GP	1
Cousin	1			Organization	1

**Table 6:** The criteria for selection and characteristics of the three types of social networks for condition management

Type of social network	Criteria for inclusion	Centrality of ties	Family role in health management	Health‐care professional role in health management	Friend role in health management
Family‐focused health network (*n* = 17)	Family members outnumbered friends and health‐care professionals	Predominantly multiple family members. For some participants, their GP was also central	Multiple family members had significant roles in supporting the individual	Health‐care professionals were important, but family members were normally consulted first	Friends were less important in management. Were a source of potential support
Friend‐focused health network (*n* = 4)	Friends outnumbered family and health‐care professionals	Friends, family and GP	Important for instrumental support for younger participants, in particular parents and siblings. For older participants, family was less relevant because of emotional and physical distance. Yet these networks were characterized by a physical, instrumental or emotional absence of family support	GP has a significant role but other health‐care professionals do not	Friends are important in providing support. Differs to family focused health network as friends are a central source of support
Health‐care professional‐focused health network (*n* = 5)	Health‐care professionals outnumbered other network members	Multiple health‐care professionals. Few family members identified	Few family members identified. Primarily partner	Very significant role. The participants referred to multiple health‐care providers including GPs, nurses and specialists	Friends were not identified as significant

1. Family‐focused network.
2. Friend‐focused network.
3. Health‐care professional‐focused network.

**Figure 3:**
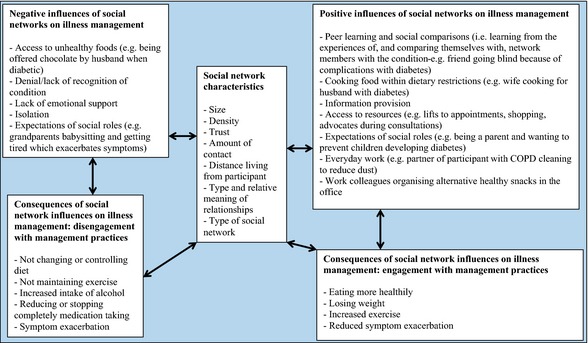
A diagram of the specific ways that social networks impact on illness management.

These categories are not meant to imply that other relationships do not have a role in (or influence on) supporting illness management (e.g. in the family‐focused network, friends do have a role), but they were relatively minor. In this study, we have used the construction of typologies as a means of enhancing analysis to summarize how relationships are described in a narrative context and have presented this using case studies of individual participants to show how the complex interactions between social networks and individual approaches to health management are interwoven (Fig. 3). These case studies were chosen as exemplars that highlighted key components of the typologies. Rather than discrete categories that occur to the exclusion of other types of support, these typologies represent a *continuum* of relationships with varying degrees of influence. Additionally, these relationships were subject to change and redefinition, rather than being static. Across the different types of networks, central network members were more likely to provide both practical and emotional support for participants. In contrast, peripheral network members were more likely to provide specific emotional or tacit support for self‐management activities. The network types depicted did not appear to reflect differences in gender or conditions (Table 1). Furthermore, participants did not report any changes in care from their general practice over the course of the study; thus, training of health‐care professionals as part of the WISE intervention was not considered to influence the networks. These results were mirrored in the main RCT results.^19^, ^27^

### Family‐focused network

Family‐focused network is the term given to networks where the main sources of support were family members. Health‐care professionals tended to be more peripheral network members who were sought for specific, task‐focused support, such as prescriptions. Health was framed as an integrated, albeit relatively minor, part of everyday life. Participants sought support from and provided support to various familial network members. When valued familial roles were threatened, these participants responded by seeking supplemental support outside of typical familial pathways (example of Tina below). Relationships with those closest, such as partners, could also be a source of tension within a network (example of Don below). Participants with this type of network typically sought support from family members for mundane and everyday tasks (such as reminders to take medicines, lifts to appointments and cooking food that supported diet control). Information and support tended to be from family members, and self‐management activities (such as exercise) were primarily performed with family members. The two case studies described below represent differential expectations of support from within the family unit and how support changed over time.

#### Positive influences of changes to familial networks on health‐management

Firstly, there was the case of Don, who was a 49‐year‐old, white male who lived with his wife, Gail, and dogs. He had diabetes, cataracts, eye problems and tendonitis. The main source of support that Don had was from Anne, his mother, who also had diabetes. He described how a perceived lack of emotional support from his wife had a negative effect on his management, which influenced when, and to what degree, resources (including support) were sought.

A:… my mum, she won't know what a carbohydrate is if it hit her in the face…we do talk about it [diabetes]… I do feel as though the only support I've got, because my wife really doesn't… doesn't really understand the feelings and things… I can talk to her but she's not one for talking to like that…

(Don's initial interview)

**Figure 4:**
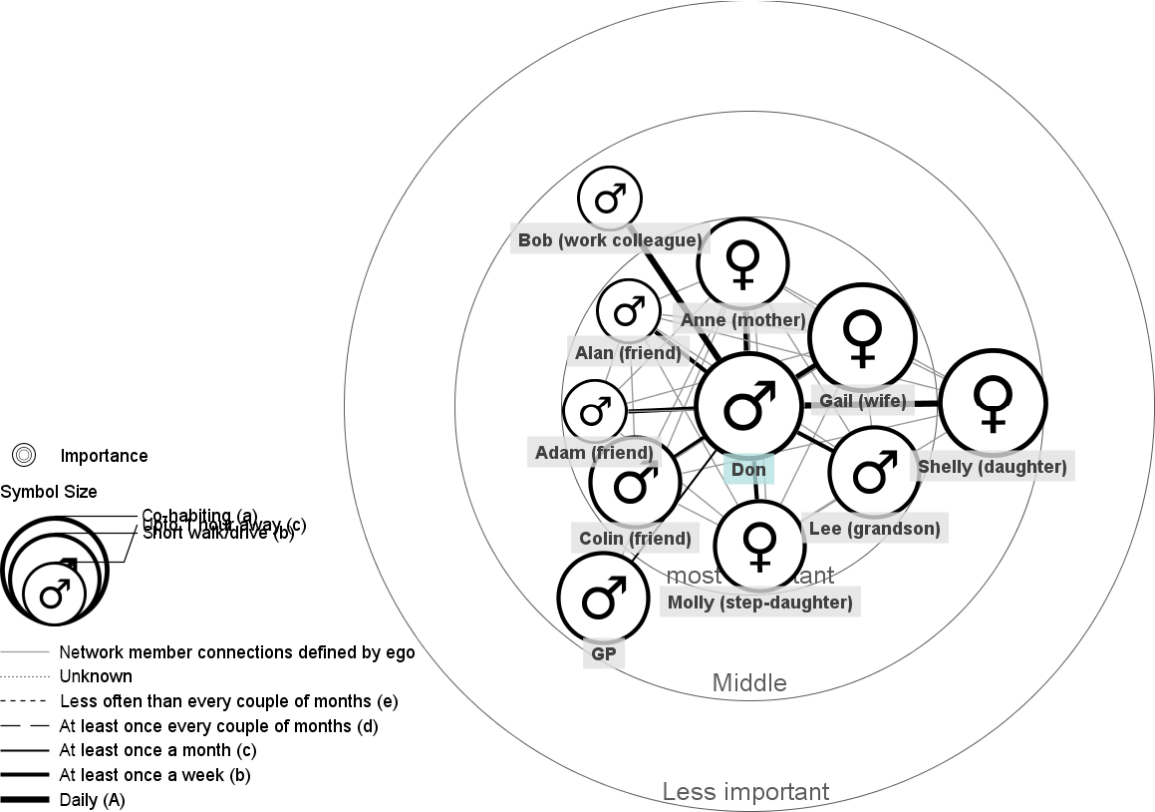
The personal health‐related social network of Don.

The loss or reconnection of ties with family members had a significant effect on the well‐being of participants, and this was reflected in the degree of engagement with illness management. Don had recently re‐established his relationship with his daughter (Shelly) after years of no contact. He also placed his grandson (Lee) in the centre of the circle, despite the fact that he had only recently established contact with him (Fig. 4). The relationship with Lee inspired Don to take better care of his health so that he would live longer. Reconnecting with his daughter and establishing a relationship with his grandson had provided Don with a valued role as a grandfather to his only grandchild and helped Don to manage the depression resulting from his ill health.

Q: …What's been the most significant change for you, sort of in the last twelve months?…

A: Well it's got to be my grandson… *it makes me want to live longer*…

(Don's final interview)

#### Negative influences of changes to familial networks on health management

**Figure 5:**
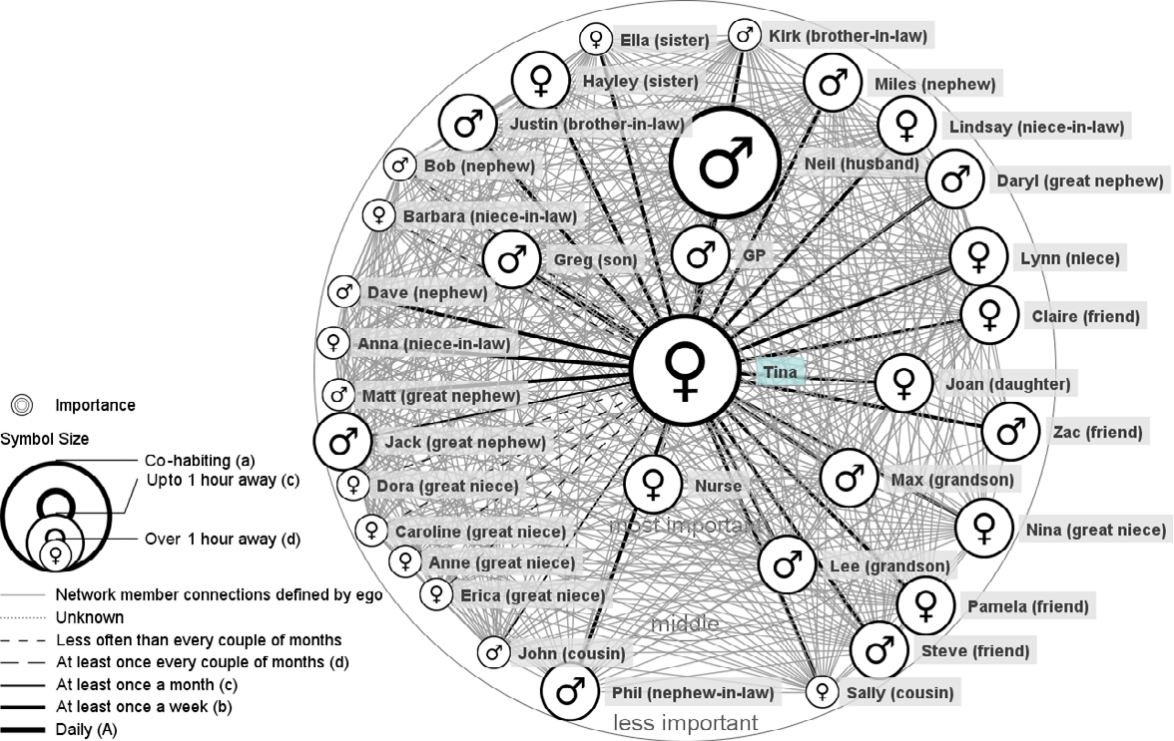
The personal health‐related social network of Tina.

The second example of a family‐focused network was Tina (Fig. 5), who was a 69‐year‐old, white female with diabetes. Over the period of this study, she developed a range of health problems, including stress incontinence, hair loss and eye infections. In the initial interview, Tina was actively involved in a singing group which she described as ‘being my escape’. However, by the final interview, she had reduced her involvement, to ensure she was not too tired to babysit her grandchildren. Tina had a large extended family, and their importance was articulated through how she described her daily routines as being shaped and restricted by being available for her family. Repeatedly, she described how much she loved them all and considered them to be equally important despite placing a great burden on her:

Maybe I do too much…I feel I can never have too many people in my life… sometimes maybe it does do me harm, because sometimes I do worry.

(Tina's final interview)

Participants with a family‐focused network tended to consider health and mundane long‐term condition management to be a minor part of everyday life, irrespective of the severity of conditions. Health was rarely in the foreground, as activities and everyday routines of their families were prioritized over their own health. For Tina, despite the effects of stress on her health resulting from her daughter's marital breakdown, her own health was not considered a priority.

I never think about it [diabetes]. I just take my tablets and, no, it's not, it's not really a priority for me…There are so many other things to think about…My family…it's just pushed at the back of my mind…

(Tina's final interview)

### Friend‐focused network

Friend‐focused networks were distinguished by the involvement of a greater number of friends and fewer family members compared to other network types. If health‐care professionals were considered significant, then it was limited to task‐specific roles (e.g. medication prescription). This type of network represents four of 26 networks. When help or advice was sought about self‐management activities, it was selectively targeted from individuals most able to provide the resources. For instance, participants would ask friends to collect medication or go swimming together to increase their exercise. This network is distinct from family‐focused networks by the apparent absence of familial support. The lack of familial support was substituted by friends who were represented as ‘fictive kin’.^28^ To illustrate a friend‐focused network in more detail, a case study of Ron will be used.

**Figure 6:**
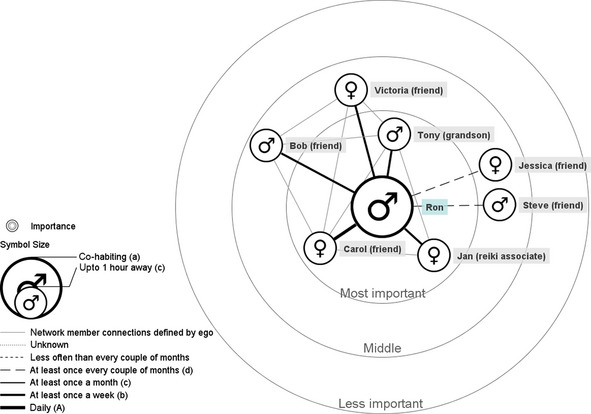
The personal health‐related social network of Ron.

Ron, an 84‐year‐old man, had diabetes, ischaemic heart disease and arthritis. Ron lived alone. The absence of family in this network is characterized by their physical loss as many of his relations were deceased. Ron's grandson, Tony, was his only living relative. Overall, Ron described being able to talk to any of his network members about health concerns and he did not include any health‐care professionals in his network (Fig. 6).

The friend‐focused networks were characterized by health being framed by individuals as a minor part of everyday life, despite all participants having multiple health concerns. For instance, Ron had undergone a knee operation and during the study period had two heart attacks; however, he did not discuss these health problems with others. This was similar to Kelleher's^29^ description of normalization, which suggested that individuals who normalize the condition change their routines to adapt, but do not complain about its effect on their life:

My health isn't a major topic for me really… Only when something like this is, you've got a chest infection, but generally I class myself as a normal healthy person… *I'm certainly no hypochondriac*.

(Ron's final interview)

Friends were an important source of meaning for his daily life. Ron spoke about the importance of his friends in terms of a selected family, particularly one close friend who he described in familial terms as ‘like another daughter’.

My closest friend, Carol… We talk every night on the phone about a quarter to ten, ten o'clock, we have ten minutes on the phone…Checking each other's alright … It's her checking up on me mainly.

(Ron's final interview)

Ron described a much closer relationship with his friends than his family. Ron spoke to his grandson out of a sense of obligation; however, despite recounting adequate support, which he valued greatly, this support was limited. At the time of the interview, swine flu was prevalent across the country. This acted as a minor epiphany,^30^ as he acknowledged the limitation of the support he had. He felt that this would have been different if his daughter was alive. This highlighted the limits of substitutability of friends over family:

I take things as it comes. But one thing that does slightly, very slightly worry me is, um, if I went down with this swine flu…I'd let me grandson know…I don't want to impose on their [friends] lives by expecting them to come and look after me…I mean if [daughter] had been alive she'd have come and lived with me …

(Ron's final interview)

### Health‐care professional‐focused network

The third health network type depicted was the health‐care professional‐focused network. Such networks were characterized by multiple health‐care professionals. Clinicians had a central role in influencing management, were consulted regularly and were predominantly considered the only legitimate people with whom to discuss health. Accounts depicted the importance of on‐going relationships with health‐care professionals and individual family members, usually a partner. Advice from health‐care professionals was prioritized. Individual family members typically provided support that was focused on specific tasks, such as preparing healthy food, and were less likely to be sought for emotional support. This type of network was represented in 5 of 26 interviews. It was not associated with the severity of conditions (i.e. these participants were no more likely than those in other types of networks to have multiple, complex conditions).

**Figure 7:**
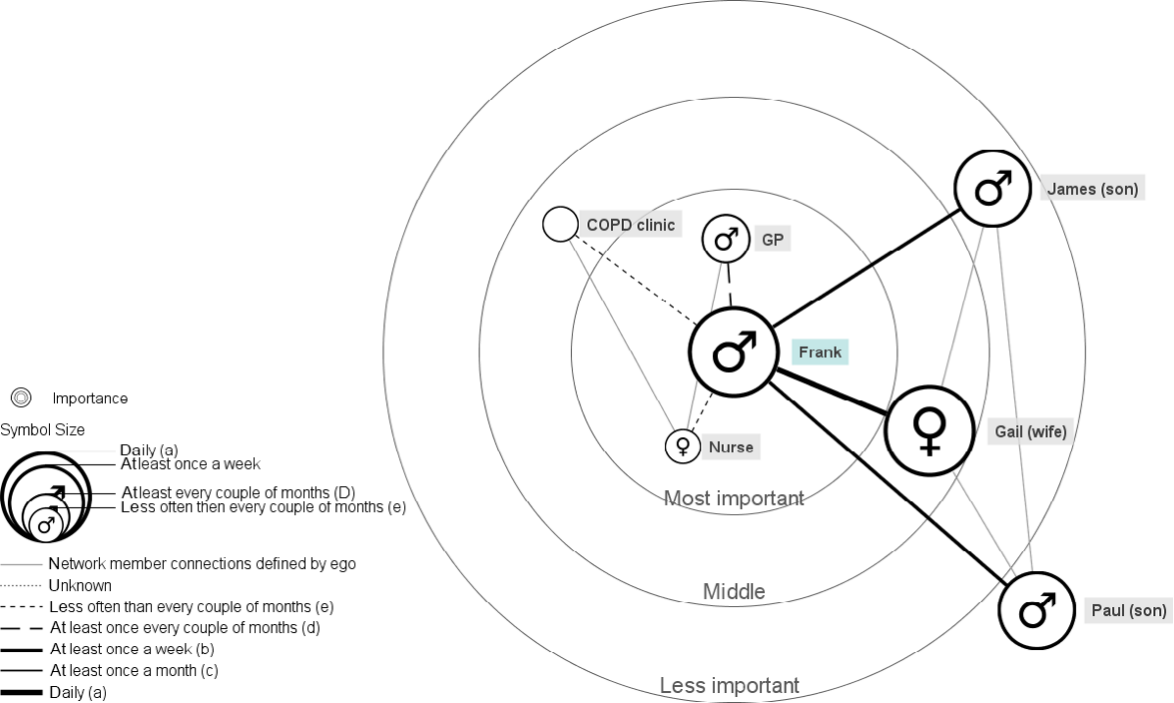
The personal health‐related social network of Frank.

To illustrate the characteristics of the health‐care professional‐focused network, we will use the case study of Frank. Despite Frank having an equal number of family and health‐related network members, his narrative depicted the importance of the information he received from health‐care professionals as having a greater impact on his on‐going management. Frank was a 65‐year‐old, white male with COPD and hypertension. He was retired and lived with his wife (Fig. 7). Being independent and taking on the role of provider for his family were important dimensions of his self‐image. Declining health was gradually challenging this image. Maintaining this independence was a source of tension with his wife, who avoided asking him to carry out tasks. In trying to protect him, she highlighted his illness, which threatened his identity and perceived usefulness to the family.

… I have not felt this bad ever, which isn't to say that I can't cope with it … *I've just got to slow down*, I can't now, I find it hard walking and talking…it is more annoying than depressing… I can cope as it is but there's not much spare capacity …I never stop in bed…*I can't say that I talk to people, but the wife knows immediately* if I am not so well, because I am quiet… But I just tend to work myself through it

(Frank's first interview)

All participants with health‐care professional‐focused networks more explicitly followed the information provided by the GP and prioritized it above other sources of information (such as family). This was in contrast to the other network types, where the participants framed information from health‐care professionals as one strand of knowledge that could be adapted to their individual context. In a health‐care professional‐focused network, participants were less likely to challenge doctors directly and instead sought other ‘legitimate’ sources (such as a nurse) for information. Frank described the way in which he sought another network member and connections between professionals to act as a bridge to the medication that he needed.

I've had a lot, a lot of chest infections… within fourteen months I had ten courses of antibiotics, so. I went to see the doctor, she [wife] said, ‘For God sake, tell them you shouldn't be like this.’ So I went…he sort of dismissed it and I come home, and he [GP] said, ‘Oh, stop worrying about it’… I went on this …COPD course and went to the see the nurse and told her.., that I should have antibiotics and she went to see the doctor…since then they clear up a lot quicker. I don't think I ever got them cleared up before…

(Frank's final interview)

### Changes to health management and social networks over time

Over the course of the year, participants described changes to their network whereby relative importance of key members changed. For example, Ron's friendship with a pharmacist was most important when he was discharged from hospital and unable to manage his medication without help. Overall, changes were found to influence how respondents managed their condition and whether or not they sought other sources of support. Changes in networks and changes in health could be mutually influential. Network changes may have wider‐ranging effects than simply the loss, or re‐establishment, of relationships. For example, such changes can prompt the reassessment of trust and meaning of existing relationships, as in the case of Don when he described re‐established a relationship with his daughter and met his granddaughter for the first time. These new relationships affected his approach to health management, from being despondent and dismissive of making changes to his lifestyle (such as diet and exercise), to actively making these changes because ‘he wanted to live longer’. However, for some participants, changes in health or social networks had little or no impact on the way in which they managed their conditions.

## Discussion

This study has empirically examined the social networks implicated in long‐term condition management. Analysis highlights three types of networks (family focused, friend focused, and health‐care professional focused) that are relevant to understanding the management of long‐term conditions. Each network type represented the way participants approached condition management and where resources (such as information or support) were sought. For instance, participants with health‐care professional‐focused networks were more likely to initially go to the doctor to seek information. Participants with family‐focused networks would discuss health problems primarily with family members. The social network types represent the context in which health management and illness practices, such as medication management, are integrated into everyday lives. Social networks influenced condition management by influencing the relative meaning of managing conditions. This, in turn, was found to shape individual prioritization of management practices (e.g. diet control, exercise) and the extent to which they were engaged with these practices. This moves beyond existing research and self‐management definitions^1^ to examine the specific processes of support and resource provision to highlight its complex and reciprocal interaction, which influences management.

Social networks influenced condition management through a number of direct and indirect processes that could have a positive (e.g. sharing of information or lifts to appointments) or negative (e.g. expectations of roles, such as being a parent or worker) effect on individual health management. This shaped the context, time available and capacity within everyday routines for condition management. The availability of support from network members varied over time. Respondents depicted flexibility in seeking support, although this support was typically focused around core individuals constrained within implicit boundaries formulated from mutual expectations of roles. It has been proposed that weak ties act as a moral positioning of self‐management between personal agency and control in self‐management.^31^ In this study, the opportunities for extending or developing weaker ties were limited. Thus, the presumption within the wider literature on social networks that there is strength in weak ties^32^ and that they have been identified as functional in other areas of social life (e.g. access to children's education^33^) might not apply to chronic illness management.

Larger networks could have a detrimental effect on condition management if relationships were considered additional work (e.g. providing support to others). This has implications in considering the relevance of measuring social networks quantitatively, as relationships were complex, often with positive and negative components. Critical moments, both positive and negative, occurred as a result of the influence of network members. These critical moments tended to have a greater influence on health in the family‐ and friend‐focused networks. Whilst many assumptions have been made regarding the influence of families on self‐management support, this has received little empirical investigation.^7^, ^31^ This approach has enabled us to understand how these relationships interact and influence an individual's orientation to condition management in community settings. These findings could be used in practice to identify whether patients have the practical and/or emotional support they need to support their self‐management priorities, where they seek it, key points of change (e.g. bereavement of spouse or friends), and the types of support people may be most likely to engage with and find useful. For instance, the tailoring of exercise advice to patients with a family‐focused network could suggest including family and friends in plans for exercise.

Social networks that were characterized by more heterogeneous composition (especially family‐ and friend‐focused networks) had access to a wider range of resources if core network members were unable to assist them. More peripheral network members may be considered weaker ties, which, as Granovetter^32^ proposed, provide access to a larger breadth of information. This is particularly relevant when considering the impact of the different types of networks on the utilization and effectiveness of education programmes. Respondents who prioritize support from health‐care professionals and minimize support of family members may not engage with initiatives that seek to increase the role of family members. Alternatively, for respondents who sought familial support, programmes that explicitly mobilize family support may be more appropriate.^34^ Identifying these different network types helps to understand why a ‘one size fits all’ approach to education programmes and policies has only limited utility.^5^

In order for self‐management support programmes to progress, they need to move beyond an individual focus. Based on the evidence presented here, the notion of ‘self’‐management needs to evolve to reflect these broader societal influences, as focusing on the individual artificially restricts sources of support. Policies and programmes that have been developed on the concept of ‘self’ are necessarily limited, exclude important resources and may reinforce existing inequity. In other words, ‘self‐management’ may be adequate in explaining individual behaviours; however, broader policy focus and programme design to support self‐management must expand to include these complex and critical social components if it is to remain relevant and useful.

## Strengths and limitations of this study

This study has moved beyond examining the structural components of the social network to understanding the mechanisms that underpin these relationships. However, it would be incorrect to assume that social networks are static or have definite boundaries. The strength of using this approach was in illuminating nuanced relationships, which have tended to remain implicit and underexplored within previous research. The central network members were discussed in all interviews. The concentric circle network diagram was only used in the final interview as we identified after the initial interviews the potential benefit of using a tool to elicit the network structure and subtle distinctions between more peripheral social network members. By examining the structure and meaning of social networks in relation to management, this study explicitly highlighted boundaries of support. Participants were purposefully sampled to include a range of ages and different lengths of time since diagnosis. Although the index conditions were restricted to those within the RCT, they were selected because of the variable provision of formal support. Despite this, the processes of seeking support were more dependent on the type of support valued by the individual, which was unrelated to the index or presence of comorbid conditions.

## Further research

Whilst this study focused on everyday management of long‐term conditions, future research could examine how networks change at different times following diagnosis. Further research needs to identify ways in which to engage individuals with long‐term conditions who value information and support from a variety of sources. Analysis of the role of pets in supporting people with long‐term conditions has been undertaken and merits further study.^35^ Future programmes need to be able to identify and react to changing support needs (such as the onset of additional conditions) and identify gaps or absence in support. Understanding is needed as to why some people respond well to formal education programmes and others do not. Such an approach could more appropriately reflect a more socialized perspective of illness management and limitations of existing programmes. A future study could examine the acceptability of referral to broader community resources from primary care.

## Conclusion

The degree to which management practices are adopted is influenced by the social context in which they occur and shaped by key relationships, which can be represented by different types of social networks. Accounts in this study depicted processes of supplementation and substitution of support, which reflected a degree of flexibility of support. Policies and disease education programmes need to be tailored as individuals need different types of support and this will ultimately affect their utility.

## Funding

This qualitative study was funded through a National Coordinating Centre for Research Capacity Development Research Training Fellowship awarded to RM. The preparation of this article was supported by The University of Manchester Health Sciences Post‐Doctoral Research Award awarded to RM. The WISE study, from which respondents for this qualitative study were drawn, is funded by the National Primary Care Research and Development Centre, Department of Health. The views represent those of the authors and not the funders. The Department of Health had no involvement in the research process or writing of this article.

## Conflicts of interest

None to declare.
